# Development of an online resource for recruitment research in clinical trials (ORRCA)

**DOI:** 10.1186/1745-6215-16-S2-P98

**Published:** 2015-11-16

**Authors:** Nicola Harman, Shaun Treweek, Mike Clarke, Paula Williamson, Pete Bower, Carrol Gamble

**Affiliations:** 1 North West Hub for Trials Methodology Research, Liverpool, UK; 2 Health Services Research Unit, University of Aberdeen, Aberdeen, UK; 3 All-Ireland Hub for Trials Methodology, Belfast, UK

## 

Recruitment to time and target remains a common challenge in the completion of a successful clinical trial. Since 2007 there has been significant investment in infrastructure to support clinical trials in the UK but the challenge of recruitment remains. The 2013 International Clinical Trials Methodology Conference demonstrated the volume of methodological research targeting recruitment. Furthermore, a 2014 survey of UK registered clinical trials units identified “research into methods to boost recruitment in trials” as the top priority for methodological research.

Research in this area needs to be managed in a resourceful way that will enable trialists and methodologists to access and use relevant research, to review methods that have been used to evaluate recruitment strategies and to identify areas that warrant further study. The ORRCA project aims to deliver this resource by creating a web-enabled database populated with published and ongoing recruitment research. Eligible studies, identified from a search of the literature and contact with funders, will be categorised into key domains. We will include all eligible studies which report or assess recruitment strategies or interventions in the context of a clinical trial. This will include randomised comparisons and “case reports” of the specific recruitment strategy used in a clinical trial.

The key domains, developed for the web-enabled database, will be presented along with the search strategy and initial results mapped to the key domains. Areas requiring further research and prioritisation will be identified.

